# The Inflammation Response to DEHP through PPARγ in Endometrial Cells

**DOI:** 10.3390/ijerph13030318

**Published:** 2016-03-14

**Authors:** Qiansheng Huang, Huanteng Zhang, Ya-Jie Chen, Yu-Lang Chi, Sijun Dong

**Affiliations:** 1 Key Lab of Urban Environment and Health, Institute of Urban Environment, Chinese Academy of Sciences, Xiamen 361021, China; htzhang@iue.ac.cn (H.Z.); lywchen@163.com (Y.-J.C.); ylchi@iue.ac.cn (Y.-L.C.); 2 Ningbo Urban Environment Observation and Research Station-NUEORS, Chinese Academy of Sciences, Ningbo 315800, China

**Keywords:** di-(2-ethylhexyl) phthalate, endometrium, *Ishikawa* cells, inflammation, PPARγ

## Abstract

Epidemiological studies have shown the possible link between phthalates and endometrium-related gynecological diseases, however the molecular mechanism(s) behind this is/are still unclear. In the study, both primary cultured endometrial cells and an endometrial adenocarcinoma cell line (*Ishikawa*) were recruited to investigate the effects of di-(2-ethylhexyl) phthalate (DEHP) at human-relevant concentrations. The results showed that DEHP did not affect the viability of either type of cell, which showed different responses to inflammation. Primary cultured cells showed stronger inflammatory reactions than the *Ishikawa* cell line. The expression of inflammatory factors was induced both at the mRNA and protein levels, however the inflammation did not induce the progress of epithelial-mesenchymal transition (EMT) as the protein levels of EMT markers were not affected after exposure to either cell type. Further study showed that the mRNA levels of peroxisome proliferator-activated receptor gamma (PPARγ) wereup-regulated after exposure. In all, our study showed that human-relevant concentrations of DEHP could elicit the inflammatory response in primary cultured endometrial cells rather than in *Ishikawa* cell line. PPARγ may act as the mediating receptor in the inflammation reaction.

## 1. Introduction

Phthalates are a group of organic chemicals used as plasticizers in various products. Di-(2-ethylhexyl) phthalate (DEHP) is the most commonly used among them. Phthalates are widely detected in the environment and organisms including humans. Young women suffer greater exposure than men at the same age, possibly due to the more frequent use of cosmetics containing these substances [1]. The adverse effects on female reproduction have received great attention [2,3,4]. Health impacts have been reported on birth [5], gynecological diseases [6,7], and sexual function [8], however, no clear conclusion can be made about the effects of phthalates on human reproduction. Endometriosis and other endometrium-related diseases are widely studied as the health outcomes of phthalate exposure. Epidemiological studies have been widely conducted to reveal the associations between phthalate exposure and endometrium-related diseases, such as endometriosis and uterine leiomyomata [6,9,10,11,12], however, the associations between them were inconsistent. The serum concentrations of DEHP and its metabolites were significantly higher in those patients with advanced-stage endometriosis than endometriosis-free controls from a case-control study [9]. In contrast, no significant association was observed between urinary concentrations of DEHP metabolite and the risk of endometriosis in infertile Japanese women [12]. Even more, the concentration of mono-2-ethylhexyl phthalate (MEHP) was negatively related to the risk of endometriosis [10]. From the aspect of laboratory studies, DEHP exposure was found to enhance the viability of human endometrial cells [13]. To our knowledge, further understanding about the molecular toxicology remains unclear.

Inflammation was involved in the cyclical remodeling of the endometrium. Inflammatory factors play important roles in the modulation of a variety of endometrial functions [14]. An epidemiologic study from ten European countries showed that IL-1β was involved in the induction of carcinogenesis in endometriotic tissue [15].

Peroxisome proliferator-activated receptors (PPAR) play important roles in gynecological disorders [16]. They represent a highly conserved nuclear receptor family, which is related to the physiology and pathology of the female reproductive system. The protein levels of both PPARα and PPARβ subtypes were significantly higher in endometrial cancer compared to the normal control [17]. On the contrary, the expression of PPARγ was decreased in endometrial cancer and proliferative endometrium. Since the effects of DEHP could be mediated by PPAR [18], the possible role of PPAR in the effects of DEHP on the endometrium is of interest. 

Abnormality of the endometrium could lead to diseases, such as endometriosis and endometrial cancers. Phthalates are likely to act as endometrium toxicants. In this study, two kinds of cells (primary cultured endometrial stromal cells and *Ishikawa* cell line) were selected to investigate the effects of DEHP on the endometrium *in vitro*.

## 2. Materials and Methods 

### 2.1. Cell Culture and Treatment

The study was conducted strictly following the government policies and the Declaration of Helsinki, with the approval of our Institutional Review Board (Reg. no. 0103). Endometrial stromal cells (ESCs) were primarily cultured from the eutopic endometrium of endometriosis patients according to a previous study [19]. Briefly, the endometrium was separated and cells were isolated by filtration using different sizes of filters. The endometrial stromal cells were identified by vimentin antibody exposure. Human endometrial adenocarcinoma cell line (*Ishikawa*) was obtained from a standard stock culture (Cell Bank, Chinese Academy of Sciences, Shanghai, China). Both cells were cultured in DMEM/F-12 (Hyclone, Logan, UT, USA) supplemented with 10% FBS (Hyclone) under 37 °C in humidified air containing 5% CO_2_. Di-(2-ethylhexyl) phthalate (DEHP, AccuStandard, New Haven, CT, USA) was dissolved in dimethyl sulfoxide (DMSO) before addition to the cell culture. The vehicle control group contained 0.1% DMSO in the culture medium. The serial concentrations of DEHP exposure were 0, 0.2, 2, 20 and 200 μM for MTT assay. For all the other experiments, four doses (0, 0.2, 2 and 20 μM) were applied based on human population urine concentrations as indicated in the discussion section. 24 h before incubation with DEHP, the estrogen was depleted by replacing the culture media with phenol red-free DMEM/F-12 supplemented with 10% charcoal-stripped FBS.

### 2.2. MTT Assays

The proliferation of both cells was detected by a MTT (Sigma-Aldrich, St. Louis, MO, USA) colorimetric assay. The initial cell density was 6000 per well. The endpoint was set at 48 h after DEHP exposure. The optical density (OD) was measured at 550 nm using a spectraMAX M5 micro-plate reader (Molecular Devices, Sunnyvale, CA, USA).

### 2.3. RNA Isolation and SYBR Realtime RT-PCR

Total RNA was isolated from cells by RNA extraction kit (Omega Bio-Tek, Norcross, GA, USA). Then, 1 μg RNA was subjected to cDNA synthesis kit (Takara, Dalian, China) following the manufacturer’s instructions. SYBR realtime PCR reaction was performed on a Roche 480 instrument (Roche, Basle, Switzerland) with a SYBR Premix Ex TaqTM kit. All tested genes were detected with the thermal profiles as follows: an initial denaturation step at 95 °C for 30 s, then 40 cycles of 95 °C for 5 s and 60 °C for 20 s, finally analysis of dissociation curve. Glyceraldehyde-3-phosphate dehydrogenase (GAPDH) was used as the reference gene. The relative fold changes of the tested genes were analyzed by the 2^−ΔΔCt^ method [20]. The primer sequences were designed by NCBI primer blast and checked by Oligo 6 software (Appendix Table A1). The theoretical annealing temperatures were all set between 62 and 65 °C.

### 2.4. ELISA Assay

The concentrations of interleukin-8 (IL-8) in the cell culture media were determined using a commercial kit (R&D Systems, Minneapolis, MN, USA).

### 2.5. Western Blot Analysis

Whole cell lysates were extracted and then subjected to SDS-PAGE electrophoresis. Then, the protein was transferred to the nitrocellulose membrane, followed by incubation with antibodies. Immuno-reactive bands were visualized using chemiluminescence. GAPDH, E-cadherin and vimentin antibodies were all from Abcam Company (Cambridge, UK). The working solution was diluted with 1:1000 for E-cadherin, 1:2000 for vimentin, and 1:2000 for GAPDH.

### 2.6. Data Analysis

All values were presented as mean ± SE. Homogeneity of variance among the groups was tested and then data significance was verified by one-way ANOVA with least-significant-difference (LSD) methods. Significant difference was accepted as * *p* < 0.05, ** *p* < 0.01, *** *p* < 0.001 *vs.* control.

## 3. Results

### 3.1. DEHP Did Not Inhibit the Proliferation of Endometrial Cells

**Figure A1:**
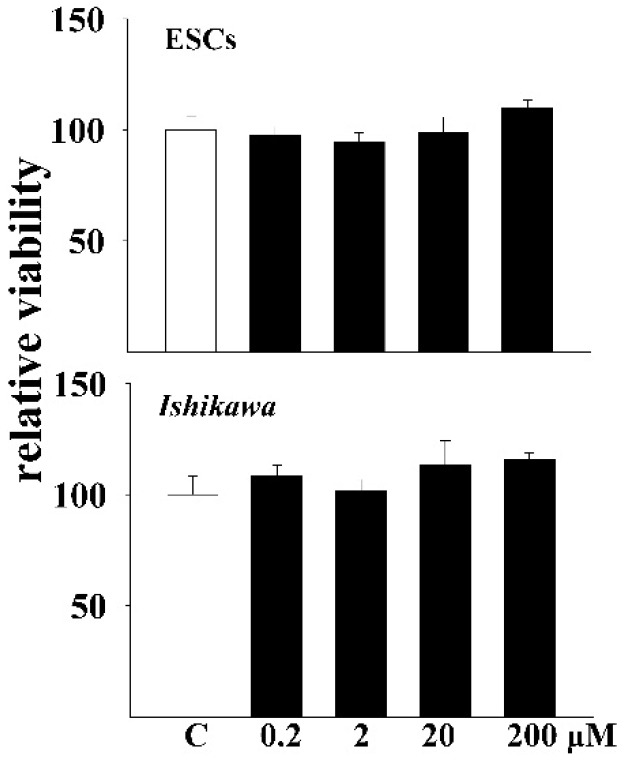
Effects of DEHP on cell proliferation after exposure for 48 h by MTT assay. The nominal concentrations of DEHP were 0.2, 2, 20, and 200 μM. Data was expressed as means ± SE.

The MTT assay was utilized to measure the effects of DEHP on the proliferation of ESCs. Results showed that DEHP did not inhibit the proliferation of the cells at doses of 0.2, 2, 20 and 200 μM (Appendix Figure A1). We also measured the proliferation of the *Ishikawa* cell line after the same treatment. Exposure to DEHP also showed no significant impact on these cells.

### 3.2. The Inflammatory Response of Cells to DEHP Exposure

**Figure 1:**
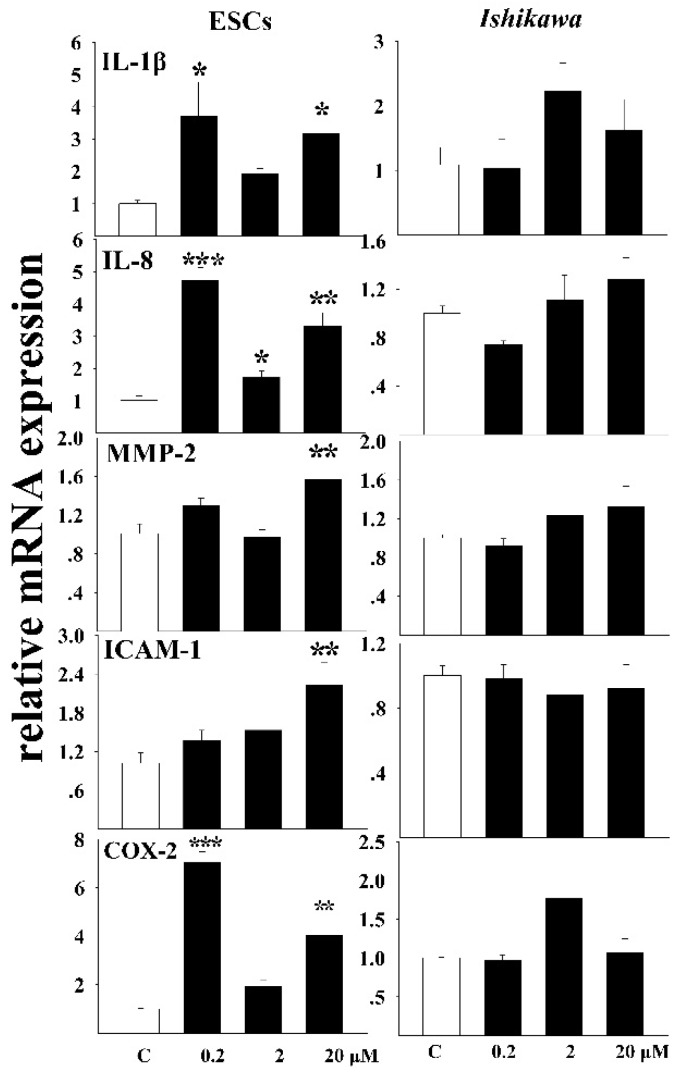
Relative mRNA expressions of inflammatory factors after DEHP exposure. Three doses (0.2, 2, and 20 μM) were used in the study. Data was expressed as means ± SE. One way ANOVA was applied to the statistical analysis, * *p* < 0.05, ** *p* < 0.01, *** *p* < 0.001.

The mRNA levels of inflammatory factors were detected both in ESCs and *Ishikawa* cells after exposure (Figure 1). The relative levels of interleukin 1β (IL-1β) were induced after exposure of ESCs to DEHP. Significant increasing was observed in both 0.2 μM (*p* < 0.01) and 20 μM DEHP exposed groups (*p* < 0.05). Interestingly, data significance was not observed after exposure to the intermediate concentration of 2 μM. The mRNA levels of IL-8 were also induced after DEHP incubation (0.2 μM, *p* < 0.001, 2 μM, *p* < 0.05, 20 μM, *p* < 0.01). In contrast, data significance was not observed for both IL-1β and IL-8 in *Ishikawa* cells. The relative amount of MMP-2 transcript was not significantly changed in both cell types except that in the 20 μM-exposed ESC cells. The expression of intercellular cell adhesion molecule-1 (ICAM1) was dose-dependently stimulated in ESCs while no obvious change was observed in *Ishikawa* cells. DEHP triggered the expression of COX-2 in ESCs. Like IL-1β, exposure to relatively lower (0.2 μM) and higher (20 μM) doses of DEHP elicited a significant increase of COX-2 mRNA transcript. No significant difference of COX-2 transcript could be observed in *Ishikawa* cells after exposure.

### 3.3. DEHP Induced the Secretion of Inflammatory Factors

**Figure 2:**
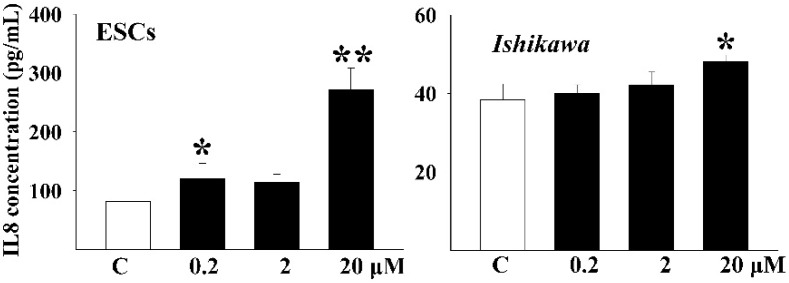
The concentration of IL-8 in the cell culture supernatant after DEHP exposure by ELISA kit. One-way ANOVA, * *p* < 0.05, ** *p* < 0.01.

The concentration of IL-8 was measured in the culture media by ELISA (Figure 2). A significant increase of IL-8 concentration was observed in the 0.2 μM (*p* < 0.05) and 20 μM (*p* < 0.01)-treated ESCs group. In *Ishikawa* cells, data significance was only detected in the 20 μM-treated group (*p* < 0.05).

### 3.4. Effects of DEHP on Epithelial-Mesenchymal Transition (EMT)

**Figure 3:**
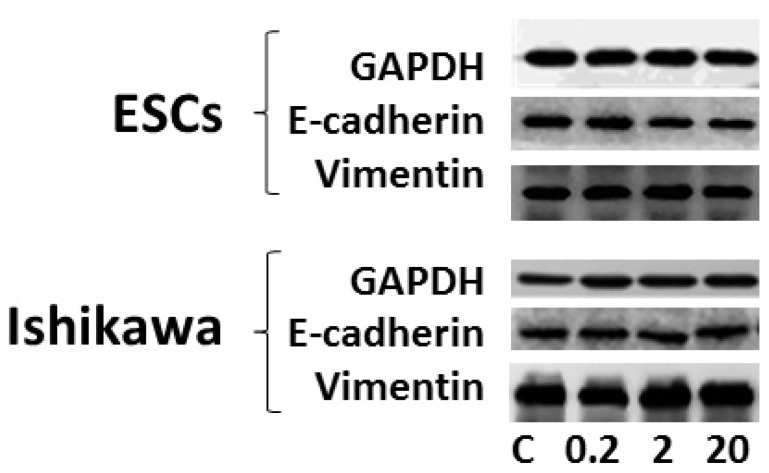
The protein levels of EMT markers after DEHP exposure determined by western blot. GAPDH was set as the reference marker.

Inflammation could induce EMT, therefore, the effects of DEHP on the expression of EMT markers were determined in both cells (Figure 3). DEHP exposure did not significantly change the protein levels of epithelial marker (E-cadherin) in both cells. The protein level of mesenchymal marker (vimentin) was also not significantly altered after DEHP exposure.

### 3.5. Effects of DEHP on the mRNA Expressions of Related Receptors

**Figure 4:**
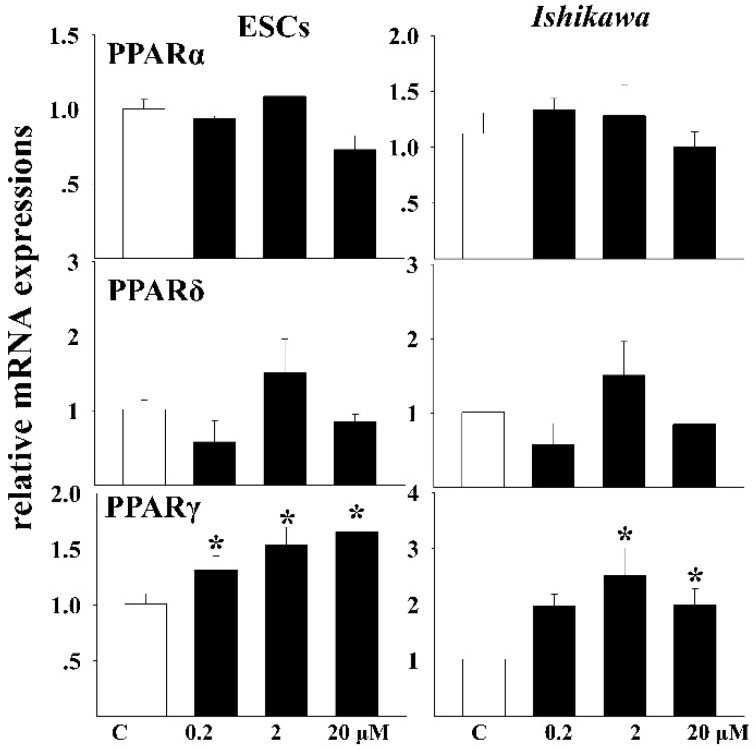
Relative mRNA expressions of PPAR receptors after DEHP exposure. One-way ANOVA, * *p* < 0.05.

The relative mRNA levels of PPAR receptors were measured after exposure in both cells (Figure 4). The same trend was observed for the response of both PPARα and PPARδ in both cells. No significant change of the mRNA levels occurred after exposure to DEHP. For the PPARγ subtype, the mRNA transcript was both significantly increased in the two cell lines. The increasing trend showed a dose-dependent relation in ESCs.

## 4. Discussion

Two kinds of cells were adopted in our study. Primary endometrial stromal cells were obtained from the endometrium. It could well represent the physiology of endometrial tissues. The *Ishikawa* cell line was cultured from a well-differentiated adenocarcinoma of the human endometrial epithelium. It has been used as a classical model in the detection of estrogenic chemicals, especially in the response of the endometrial epithelium to chemical exposure [21]. Different responses to DEHP exposure were observed between the two kinds of cells. The inflammatory reaction was more obvious in primary cultured cells than in the *Ishikawa* cell line. The results indicated that the different responses to chemical exposure between primary cultured cells and the cell line should be kept in mind.

Detected concentrations of DEHP metabolite were in the range of ng/mg urinary creatinine in general populations and the value in the highest quartile could be from tens to thousands [6,22]. Total urine DEHP concentrations surpassed 0.18 μM in half of the samples from 287 women of reproductive age in a study [10]. Three nominal doses of DEHP (0.2, 2, and 20 μM) were adopted in our study. These exposure doses could reflect real internal human exposures. Similar doses (2.56, 25.6, 256 μM or 10, 100 ng/mg) were also considered environmentally relevant in previous studies [23,24]. 

Kim *et al.* found that DEHP exposure at doses of 0.01 and 1 μM led to a significant increase of the viability of human endometrial cells [13]. In contrast, DEHP at 10 ng/mL (about 0.025 μM) did not affect the viability of endometrial cells from cows [25]. In accordance with these results, data significance was not be observed for both cells upon exposure to serial doses of 0.2, 2, 20, and 200 μM in our study. One way ANOVA (LSD method) was used in our study to compare the data significance between control, 0.2, 2, 20, and 200 μM DEHP exposed groups. Only two doses were adopted and the statistical method was not presented in the study by Kim *et al.* The difference in data analysis may account for the observed inconsistencies.

The disturbance caused by inflammation contributes to endometrial pathology [26]. *In vivo*, maternal DEHP exposure *in utero* elicited systemic inflammation in offsprings [27]. Our results showed that exposure to DEHP could stimulate the inflammatory response in primary cultured ESCs. Other *in vitro* studies also validated the stimulatory effects on inflammation. DEHP triggered the release of IL-1β in macrophages [28,29]. COX-2 was overexpressed in endometrial cancers and its inhibitors are regarded as potential therapeutics [30]. In our study, the stimulation of COX-2 expression was also observed. This showed that COX-2 also responded to the chemical exposure.

An inflammatory microenvironment could induce the development of epithelial-mesenchymal transition (EMT) [31]. EMT is detected as a decrease of epithelial marker and an increase of mesenchymal marker. During the EMT process, epithelial cells lose their polarity and acquire the mesenchymal phenotype for invasion. EMT was reported to be critical for the development of cancer [32]. Both endogenous and exogenous parameters could affect the EMT process. A previous study showed that chemical pollutants could induce the EMT [33]. In contrast, no significant change of either epithelial or mesenchymal markers was observed at the protein levels as indicated in Figure 3. This indicated that exposure to DEHP did not induce EMT in either *Ishikawa* cells or primary cultured endometrial stromal cells. Besides, the mesenchymal and epithelial marker proteins were both detectable in primary ESCs. This double-positive phenotype might be the sign of the EMT process in endometrial tissues. The similar phenotype was also discovered in other types of cancer [34].

To reveal the possible receptors that mediate the toxicity of DEHP, all the three types of PPAR was included in our study. PPAR is a subset of the nuclear hormone receptor superfamily which plays important regulatory roles in immunity and inflammation [35]. Altered expression of PPAR occurs in endometrial cancer [17]. It is reported that PPARγ was widely involved in the gynecologic disorder [16]. In our study, only the gamma subtype of PPAR showed changed expression after DEHP exposure. An increased level of PPARγ was detected after exposure in both kinds of cells. DEHP may exert its toxicity through the mediation of PPARγ, however, binding experiments are needed to validate this hypothesis.

## 5. Conclusions 

In all, primary cultured cells and a cancer cell line were both used to investigate the effects of DEHP on endometrium-related diseases. Inflammatory induction was observed after exposure to DEHP at equal concentrations in human plasma. DEHP may thus promote the development of endometrium-related diseases through induction of inflammation. This result could help elucidate the association between phthalate exposure and endometrium-related diseases.

## Appendix

**Table A1:** Primers used in the quantitative RT-PCR analysis.

Gene	Nucleotide Accession Number	Primer Sequences Used for qRT-PCR (5’ to 3’)
Glyceraldehyde-3-phosphate dehydrogenase(GAPDH)	*NM_002046*	F: GGAGAAGGCTGGGGCTCAT
		R: TGATGGCATGGACTGTGGTC
Interleukin 1 beta (IL-1β)	*NM_000576*	F: GGACAAGCTGAGGAAGATGC
		R: TCGTTATCCCATGTGTCGAA
Interleukin (IL-8)	*NM_000584*	F: GCATAAAGACATACTCCAAACC
		R: ACTTCTCCACAACCCTCTG
Intercellular cell adhesion molecule-1 (ICAM-1)	*J03132*	F: CCGGAAGGTGTATGAACTGA
		R: GGCAGCGTAGGGTAAGGTT
Matrix metalloproteinase-2 (mmp-2)	*NM_001127891*	F: TGACGGTAAGGACGGACTC
		R: ATACTTCACACGGACCACTTG
Cyclooxygenase-2 (COX-2)	*M90100*	F: GCCTGAATGTGCCATAAGACTGAC
		R: AAACCCACAGTGCTTGACACAGA
Peroxisome proliferator-activated receptor alpha (PPARα)	*NM_001001928*	F: ACGGAAAGCCCACTCTGCCCCCTCTC
		R: CTTGTCCCCGCAGATTCTACATTCG
Peroxisome proliferator-activated receptor beta/delta (PPARδ)	*NM_001171818*	F: GGCCATCATTCTGTGTGGAGAC
		R: CAGGATGGTGTCCTGGATAGC
Peroxisome proliferator-activated receptor (PPARγ)	*NM_138712*	F: GCCATTTTCTCAAACGAGAGTCAGC
		R: CCACGGAGCTGATCCCAAAGTT
